# Blow fly stable isotopes reveal larval diet: A case study in community level anthropogenic effects

**DOI:** 10.1371/journal.pone.0249422

**Published:** 2021-04-14

**Authors:** Charity G. Owings, William P. Gilhooly, Christine J. Picard

**Affiliations:** 1 Department of Biology, Indiana University-Purdue University Indianapolis, Indianapolis, Indiana, United States of America; 2 Department of Earth Sciences, Indiana University-Purdue University Indianapolis, Indianapolis, Indiana, United States of America; Universidad de Cádiz, Facultad de Ciencias del Mar y Ambientales, SPAIN

## Abstract

Response to human impacts on the environment are typically initiated too late to remediate negative consequences. We present the novel use of stable isotope analysis (SIA) of blow flies to determine human influences on vertebrate communities in a range of human-inhabited environments, from a pristine national park to a dense metropolitan area. The refrain “you are what you eat” applies to the dietary isotope record of all living organisms, and for carrion-breeding blow flies, this translates to the type of carcasses present in an environment. Specifically, we show that carnivore carcasses make up a large proportion of the adult fly’s prior larval diet, which contrasts to what has been reportedly previously for the wild adult fly diet (which consists of mostly herbivore resources). Additionally, we reveal the potential impact of human food on carcasses that were fed on by blow flies, underscoring the human influences on wild animal populations. Our results demonstrate that using SIA in conjunction with other methods (e.g., DNA analysis of flies) can reveal a comprehensive snapshot of the vertebrate community in a terrestrial ecosystem.

## Introduction

A holistic view of community structure, encompassing primary producers to consumers, is required to better characterize the impacts of global climate change and anthropogenic effects on ecosystems to inform mitigation measures [1]. However, traditional vertebrate diversity surveillance techniques, such as transect sampling and trapping, can be labor-intensive and require considerable expertise [2–4]. Active sampling of individuals misses other taxa that may be important in an organism’s food web, as well as its ecosystem function. More popular minimal-effort surveillance techniques, like camera trapping, also come with their own disadvantages and suffer from sampling biases that overlook important dimensions of the community. Camera trapping specifically may miss nearby taxa outside of the camera’s field of view and is often biased towards capturing images of larger animals [5–7]. Additionally, such methods limit the number of taxa or guilds that can be evaluated simultaneously, requiring implementation of several methods (increasing sampling effort and cost) for total community assessment.

The shortcomings of traditional vertebrate surveillance methods can be improved with the use of necrophagous insects. Blow flies (Diptera: Calliphoridae) develop on animal carcasses as larvae and then continue to “sample” (i.e., taste) vertebrate resources (e.g., carcasses, feces) during their adult lives. These natural behaviors can then be used by scientists to indirectly monitor vertebrate communities [8, 9]. Previous work has indicated that DNA from such vertebrate resources can be extracted from the blow fly guts and sequenced for species identification [10–12]. Blow fly-derived vertebrate richness assessments have been determined to out-perform traditional surveillance methods [11, 13], and as an added bonus, they require minimal field-sampling time with increased detection potential (e.g., in 240 min, 26–43% of common mammal species in an environment can be detected using blow flies [12]). Furthermore, a vertebrate-specific fecal test (targeting vertebrate urobilinoids) can determine if the resource fed on by the adult fly was feces vs. carrion [14], giving more information to the researcher about whether the vertebrates assessed were alive or dead at the time of sampling.

**Fig 1:**
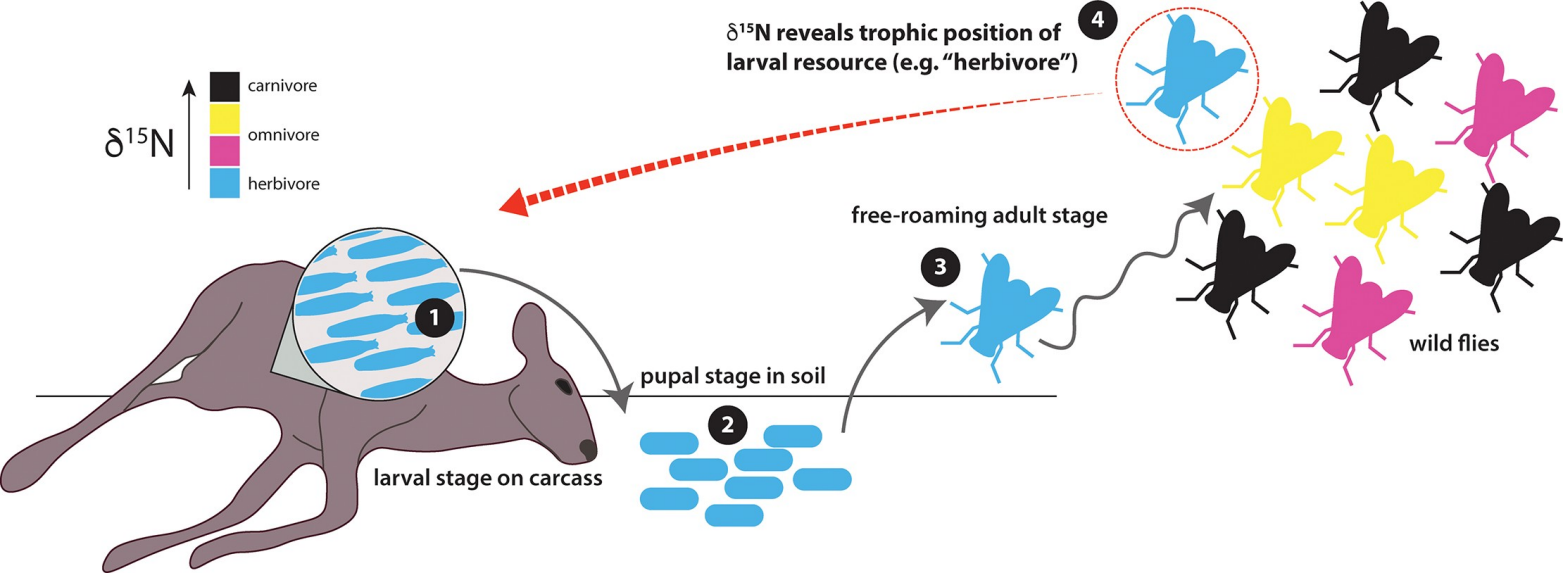
Schematic illustrating the concept for determining trophic position of the carcass. 1) Blow fly larvae develop on a carcass (e.g., a deer) by consuming the animal’s tissue. 2) Larvae leave the carcass in order to pupate in the soil. 3) Adult blow flies emerge from puparia approximately 2 weeks after initial pupation and begin the free-roaming stage of their life cycle. 4) Analysis of a random adult blow fly can reveal the trophic position of the animal on which it developed as a larva. Different colors represent different carcass trophic positions and thus the variable isotopic signatures present within wild populations of adult blow flies. In the example illustrated here, the adult blow fly retains the δ^15^N value of the carcass (“herbivore”) from its larval stage.

Here we present a complementary method to DNA analyses by using nitrogen (δ^15^N) and carbon (δ^13^C) stable isotopes of adult blow flies to understand ecosystem vertebrate trophic structure. Molecular DNA analysis of fly guts can only give information about recent resources the fly has visited, not the type of carcass used for larval development. In contrast, stable isotope signatures provide a record of resources utilized over a longer time period with the knowledge that the resource was indeed deceased. Knowing the prior development substrate of a fly is particularly important for gaining a more comprehensive view of the vertebrate community in an area, as DNA profiles from flies may have originated from resources other than carcasses (e.g., feces). The adult fly’s nitrogen isotopic composition, when calibrated for fractionation effects during feeding, can indicate the trophic level of the individual carcass that the fly used for its larval development [15, 16] (Fig 1). We propose that by employing this method in conjunction with gut content sequencing (which approximates community diversity at the time of sampling), scientists will be able to track faunal distributions of the recent past (i.e., two to three weeks before sampling, when the life cycle of the fly began).

Stable isotope analysis (SIA) is an important tool in trophic food web ecology. Animals incorporate isotopes from food into their own tissues, which provides a record of the animal’s diet over time [17]. Nitrogen isotopes are associated with the trophic position of a consumer due to the preferential incorporation of ^15^N into tissues, leading to an approximate 3‰ increase in δ^15^N compared to the consumer’s diet [18, 19]. However, this generalized enrichment value has been challenged, as isotopic variation among animal taxa can be significant, and additionally, the most relevant variation actually occurs in the trophic fractionation [20, 21]. Similarly, dietary carbon isotopes are associated with the source of carbon in an animal’s diet. Carbon at the base of the food web originates from primary producers (e.g., plants, phytoplankton, algae), which exhibit different ranges of carbon isotope fractionation based on the photosynthetic pathways (e.g., C_3_ or C_4_) used to fix CO_2_ [18, 22]. Typically, the δ^13^C value of the carbon source becomes incorporated in the tissues of consumers with little fractionation (within 1‰), though the extent of fractionation can be variable for different taxa.

The usefulness of stable isotope ecology in entomological studies has increased over the last decade [23]. Though few and far between, studies involving necrophagous blow flies have revealed important ecological information on how scavengers contribute to an ecosystem. Previous ecological studies have shown that blow flies can be used to estimate the fate of nutrients and energy entering an ecosystem, as >80% of adult blow flies (*Calliphora* spp.) reared from salmon runs (estimated at 4–7 million larvae per watershed) exhibited salmon-based δ^13^C and δ^15^N values [16]. Furthermore, blow flies and other necrophagous insects show a reliable trend in isotopic signatures that tracks with seasonal and spatial changes in salmon breeding and die-offs [24]. More recently, researchers have realized the forensic significance of blow fly SIA. Bernhardt et al. (2017) analyzed isotopic fractionation in the green bottle blow fly (*Lucilia sericata*) to determine if SIA could discriminate between humans and other animals as larval substrates and found that the human trophic position cannot be deconvoluted from animals occupying the same position [15]. Additionally, Matos et al. (2018) found that although essential amino acids do not vary throughout the life stages of blow flies, non-essential amino acids become more depleted in ^13^C in the adult fly compared to its larval substrate, which could be utilized by forensic entomologists when making time since death estimations with entomological evidence [25].

At the intersection of decomposition ecology and conservation biology is the necrophagous blow fly, which, we propose, can be used as an unbiased sampler of its environment. The purpose of this study was to test the utility of using blow fly SIA to infer the trophic level structure of dead vertebrates in an area the size of a national park, which currently can only be done with traditional surveying methods and molecular analysis. However, in order to draw any meaningful conclusions from “wild” isotopic data, it was imperative to develop baseline, species-specific datasets for comparative purposes [19]. One of our objectives was to determine if the predictable fractionations for δ^15^N and δ^13^C expected from previous food web studies (3‰ for δ^15^N, ± 1‰ for δ^13^C) holds true for the black blow fly, *Phormia regina* (Meigen) (Diptera: Calliphoridae). *Phormia regina* is one of the most common and ubiquitous blow flies in North America, and it has been previously investigated as an environmental DNA sampler [4]. Furthermore, we investigated the systematic offset in δ^15^N and δ^13^C between the adult fly and its larval diet, and whether this fractionation (Δδ = δ_fly_− δ_larval resource_) can be affected by other variables, such as the preservation method of the fly, adult feeding events, and age and sex of the fly.

## Results

As the overall goal of this study was to assess carcass trophic levels using SIA of adult blow flies, it was necessary to perform a series of experiments informing our analysis of wild-caught flies. First, it was important to determine if our field preservation method would significantly affect isotopic signatures in wild-caught flies (first experiment). In order to extract trophic structure from the wild fly isotope compositions, the fractionation effects were determined in controlled feeding scenarios (second experiment) as a basis for calculating the isotopic composition of the dietary sources. Finally, there was a need to understand how additional adult feeding (third experiment) and aging of the fly (fourth experiment) impacted isotopic fractionation values. All experiments were vital for determining how to use wild fly isotope values for carcass trophic level assessment in natural landscapes.

### Effect of preservation

It was important to address the practicality of this method, therefore, we determined if isotopic composition effects could be introduced during sample preservation as field methods differ from laboratory-based methods. We assessed this potential impact by comparing samples stored in ethanol (a typical field-based preservation method) and the effect of freezing specimens at -20°C (a typical lab-based preservation method). There was no significant difference in δ^15^N or δ^13^C between the two preservation methods (*P* > 0.05 for both). Furthermore, there were no significant differences between male or female fly isotope compositions observed in either preservation method (Table 1).

**Table 1:** Summary of isotopic data from the preservation experiment.

Preservation Methods	Sex	δ^15^N	δ^13^C
Ethanol	Male	8.78 ± 0.63	-20.00 ± 0.64
	Female	8.39 ± 0.62	-20.97 ± 0.47
Frozen	Male	7.70 ±0.62	-20.36 ± 0.45
	Female	6.50 ± 0.76	-20.46 ± 1.25

Mean (± standard deviation) δ^15^N and δ^13^C isotopic values from male (N = 10 per treatment) and female (N = 10 per treatment) flies killed via ethanol of freezing.

### Persistence of larval diet isotopic signature

**Fig 2:**
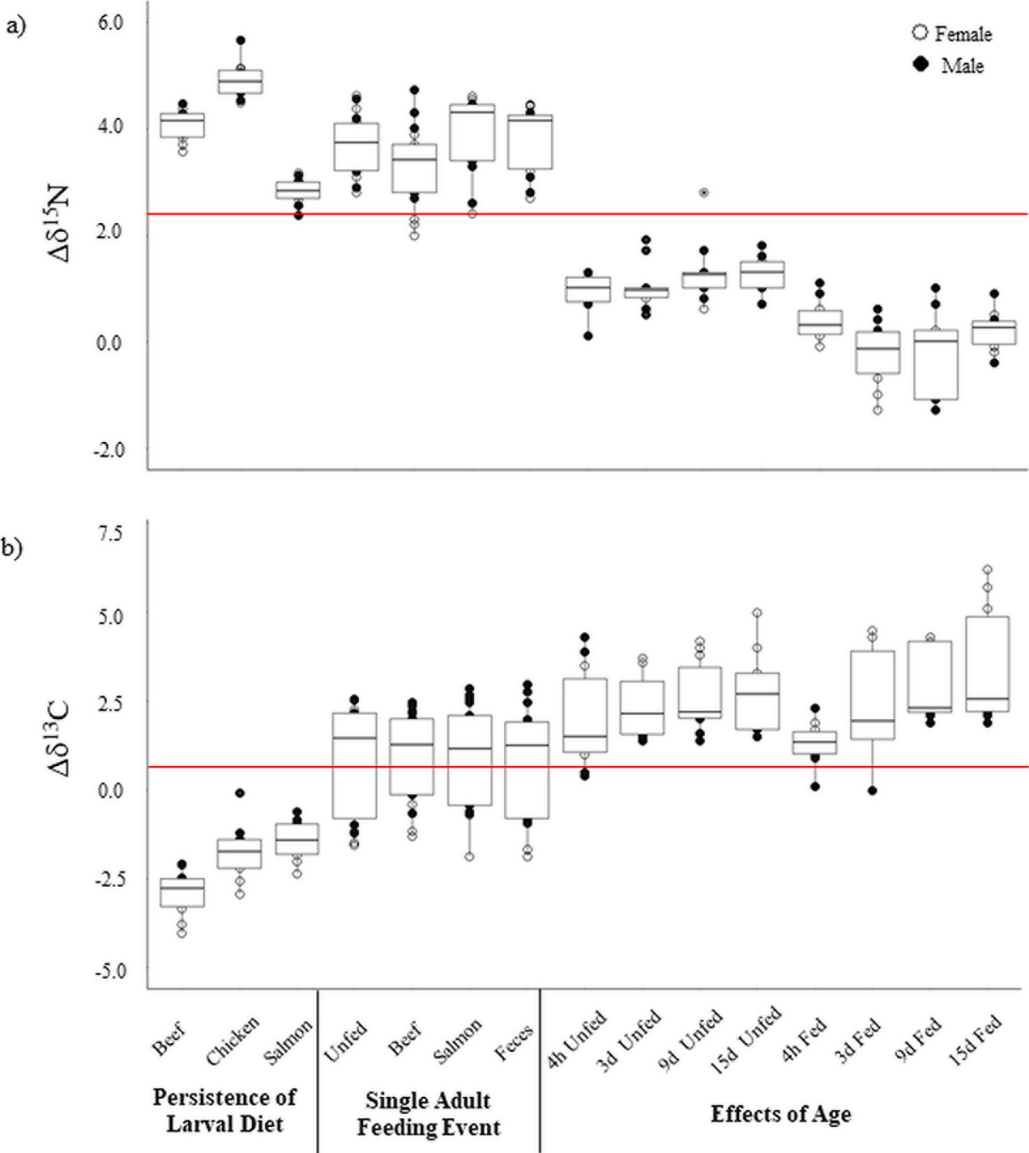
Boxplots summarizing the fractionation values for all experiments. Mean nitrogen fractionation (Δδ^15^N; panel A) and mean carbon fractionation (Δδ^13^C; panel B) are illustrated for each treatment across experiments. Open circles represent females and closed circles represent males. The red line indicates the mean value for both Δδ^15^N and Δδ^13^C across all experiments.

**Fig 3:**
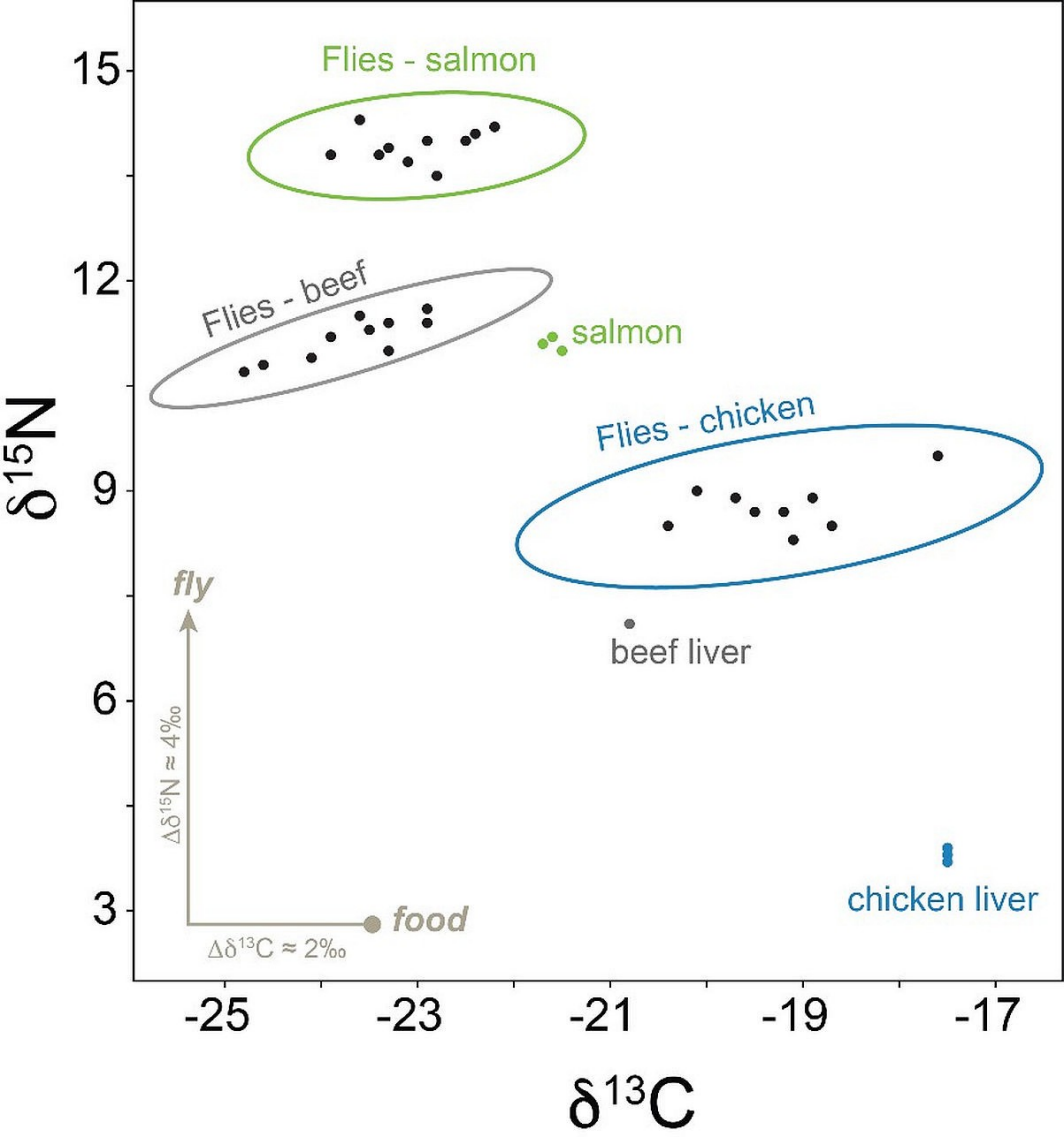
Scatterplot of δ^15^N and δ^13^C values for the persistence of larval diet experiment. Isotope relationships observed in this experiment demonstrate the possible range of fractionations during larval uptake. Circled clusters of data represent adult flies that were reared on their respective larval diets: chicken liver (blue), beef liver (grey), or salmon filet (green).

The main premise of diet analysis using stable isotopes is that an animal’s isotopic signatures reflect that of its diet, with different diets producing different isotopic values in the consumer. However, the overall deviation (= fractionation) from diet to consumer should remain relatively stable for a given organism. Therefore, we wanted to test the hypothesis that different larval diets produce similar fractionation patterns among adult blow flies, regardless of differences in their raw isotopic values. This is important to investigate in order to provide a reliable method of determining the larval diet from wild-caught adult blow flies. Adult flies exhibited systematic trophic level offsets in δ^15^N and δ^13^C relative to their respective larval resources (beef liver, chicken liver, or salmon filet), with an overall 3.9‰ increase in δ^15^N and a 2.0‰ decrease in δ^13^C across all treatments (Table 2, Figs 2 and 3). The MANOVA revealed a significant effect of treatment (*P* < 0.001) and sex (*P* < 0.001) on δ^15^N and δ^13^C fractionation together. A post-hoc ANOVA showed a significant impact of treatment (*P* < 0.001) on both δ^15^N and δ^13^C fractionations and sex for δ^13^C (*P* < 0.001), yet there were no significant interaction effects. Specifically, flies fed salmon exhibited significantly lower nitrogen isotope fractionations compared to flies fed other animal tissues, and females tended to have lower δ^13^C values compared to males. In all treatments, female *P*. *regina* exhibited nearly identical δ^15^N compared to males but were always lower in δ^13^C by approximately 1.0‰.

**Table 2:** Summary of isotopic data from feeding experiments.

					Fractionation (‰)	
Experiment	Larval Diet	Adult Diet	Other	Sex	Δδ^15^N	Δδ^13^C
Persistence of Larval Diet Isotopic Signatures	Beef	None	None	Male	4.2 ± 0.2	-2.4 ± 0.3
				Female	3.9 ± 0.3	-3.4 ± 0.5
				*Overall*	*4*.*1* ± *0*.*3*	*-2*.*9* ± *0*.*7*
	Chicken	None	None	Male	5.0 ± 0.4	-1.2 ± 0.7
				Female	4.9 ± 0.3	-2.4 ± 0.4
				*Overall*	*4*.*9* ± *0*.*4*	*-1*.*8* ± *0*.*8*
	Salmon	None	None	Male	2.8 ± 0.3	-1.0 ± 0.3
				Female	2.9 ± 0.2	-1.9 ± 0.4
				*Overall*	*2*.*8* ± *0*.*2*	*-1*.*4* ± *0*.*6*
Effects of Single Adult Feeding Event	Beef	None	None	Male	3.8 ± 0.5	1.2 ± 1.5
				Female	3.6 ± 0.6	0.4 ± 1.5
		Beef	None	Male	3.4 ± 0.7	1.3 ± 1.2
				Female	3.1 ± 0.8	0.4 ± 1.1
		Salmon	None	Male	3.8 ± 0.7	1.4 ± 1.5
				Female	4.0 ± 0.9	0.4 ± 1.3
		Feces	None	Male	3.8 ± 0.6	1.3 ± 1.6
				Female	3.9 ± 0.7	0.3 ± 1.4
				*Overall*	*3*.*7* ± 0.7	*1*.*4* ± 0.8
Effects of Age	Salmon		4h	Both	1.0 ± 0.4	2.0 ± 1.4
			3d	Both	1.1 ± 0.9	2.4 ± 0.9
		None	9d	Both	1.4 ± 0.6	2.6 ± 1.0
			15d	Both	1.3 ± 0.3	2.8 ± 1.2
				*Overall*	*1*.*2* ± *0*.*5*	*2*.*4* ± *1*.*2*
		Chicken	4h	Both	0.5 ± 0.4	1.3 ± 0.6
			3d	Both	-0.2 ± 0.6	2.3 ± 1.8
			9d	Both	-0.2 ± 1.1	3.0 ± 1.1
			15d	Both	0.3 ± 0.4	3.5 ± 1.7
				*Overall*	*0*.*1* ± *0*.*6*	*2*.*5* ± *1*.*5)*

Mean (± standard deviation) of isotopic fractionation factors (Δδ = δ_fly_− δ_larval resource_) of flies from various adult feeding treatments relative to their larval resource.

### Effect of single adult feeding event

As blow flies may feed on various carrion and fecal resources during the adult portion of their lives, it was also important for us to determine if such feeding significantly impacts isotopic fractionation from larval diet. This was particularly important for us to investigate as we needed to determine if the larval dietary signature is obscured by isotopic integration of the adult fly diet. All flies exhibited consistent trophic effects relative to their larval resource (beef liver) by an average fractionation of 3.7‰ in δ^15^N and 1.4‰ in δ^13^C (Table 2, Fig 2) when fed as adults. The MANOVA revealed a significant sex effect on δ^15^N and δ^13^C fractionation together. Post-hoc ANOVA’s revealed a weakly significant effect of feeding treatment on δ^15^N fractionation (*P* = 0.041), with fractionation values for adult flies fed beef liver being slightly lower than all other groups. Sex was the only significant factor for δ^13^C fractionation data (*P* = 0.009), with females consistently exhibiting lower fractionation than males.

### Time series

Finally, we wanted to determine if the age of the fly impacts its fractionation values from its larval resource. In laboratory colonies with sufficient resources, blow flies can live on average about 25 d [26], though this is likely reduced in nature when resource availability is unpredictable and the threat of predation is high. Depending on the organism, stable isotope composition may vary as diets change over time or ontogenetic effects, therefore, it was important to determine if the larval signature was retained in the adult fly over a majority of the typical blow fly lifespan. This is important to investigate as there is currently no reliable method of aging adult blow flies, therefore the age of flies collected in the wild is unknown. The exploratory MANOVA revealed several significant effects on δ^15^N and δ^13^C fractionation together: time (*P* < 0.001), feeding treatment (*P* < 0.001), sex (*P* < 0.001), time-treatment interaction (*P* = 0.036), time-sex interaction (*P* = 0.005), and treatment-sex interaction (*P* = 0.031). A post-hoc ANOVA revealed that feeding treatment significantly impacted δ^15^N (*P* < 0.001), whereas an interaction between time and treatment was slightly significant (*P* = 0.043). The ANOVA also showed that time, an interaction between time and treatment, an interaction between time and sex, and the interaction between treatment and sex were significant (all *P* < 0.05).

The nonparametric analyses for the Time Series experiment indicated an overall significant effect of feeding treatment on δ^15^N fractionation (*P* < 0.001), with overall lower values obtained for the fed flies. However, there were no time or sex effects on δ^15^N fractionation (both *P* > 0.05). For δ^13^C fractionation, treatment was not significant (*P* > 0.05), but time and sex were (*P* < 0.001 for both), with a slight increase in δ^13^C fractionation over time and overall higher values for females compared to males. When analyzing each feeding treatment separately, no significant time or sex effects were observed on δ^15^N fractionation in either fed or unfed flies. However, δ^13^C fractionation in fed and unfed flies was significantly affected by sex (*P* < 0.010), with females showing higher δ^13^C fractionation compared to males. Additionally, age significantly impacted δ^13^C fractionation in fed adult flies (*P* < 0.001), with older flies exhibiting significantly greater fractionation compared to younger flies (*P* < 0.001; Table 2).

### Urban and national park samples

Wild fly δ^15^N values ranged from 5.7 to 14.8‰ and δ^13^C ranged from -29.2 to -19.2‰. Raw data distributions grouped roughly according to region (urban, Smokies, Yellowstone). Flies collected in the urban environment exhibited significantly higher δ^15^N values compared to the other two environments (*P* < 0.001), with more similar δ^15^N values seen between the two national parks. In terms of δ^13^C, the values of Yellowstone flies were significantly lower than flies from the urban environment and from the Smokies (*P* < 0.001). In order to determine the carcass trophic positions derived from wild fly isotopic signatures, all fractionation data for female flies from all experiments (since only female flies were analyzed from the wild sampling collections) was averaged for both δ^15^N and δ^13^C, giving a mean of 2.1‰ enrichment in δ^15^N and 1.4‰ depletion in δ^13^C. As these fractionation values aligned with similar studies of blow flies [15], the average fractionation value of 2.1 was subtracted from wild fly δ^15^N values and the average value of 1.4 was added to δ^13^C values, resulting in carcass δ^15^N values ranging from 3.6 to 12.8‰ and carcass δ^13^C ranging from -30.6 to -20.6‰.

Based on the isotopic composition of animal tissues gathered from the literature, three conservative trophic categories were statistically differentiated based on δ^15^N values: herbivore, carnivore, and fish (Table 3). As an overlap did exist between herbivores and carnivores, an “herbivore/carnivore” category was also included to account for when the two trophic levels cannot be differentiated. Though fish do not technically represent a true trophic position, their high δ^15^N values are different enough from all other vertebrates that they were designated as their own category for the purposes of this study. Omnivores were excluded as a definitive category as their isotopic values (both δ^15^N and δ^13^C) overlapped with several other categories and could therefore not be statistically differentiated.

**Table 3:** Ranges of δ^15^N values for each vertebrate trophic position obtained from the literature review.

Trophic Level	δ^15^N
**Herbivore**	<5.7
**Herbivore/Carnivore**	5.8–5.9
**Carnivore**	6.0–10.9
**Fish**	>11.0

**Fig 4:**
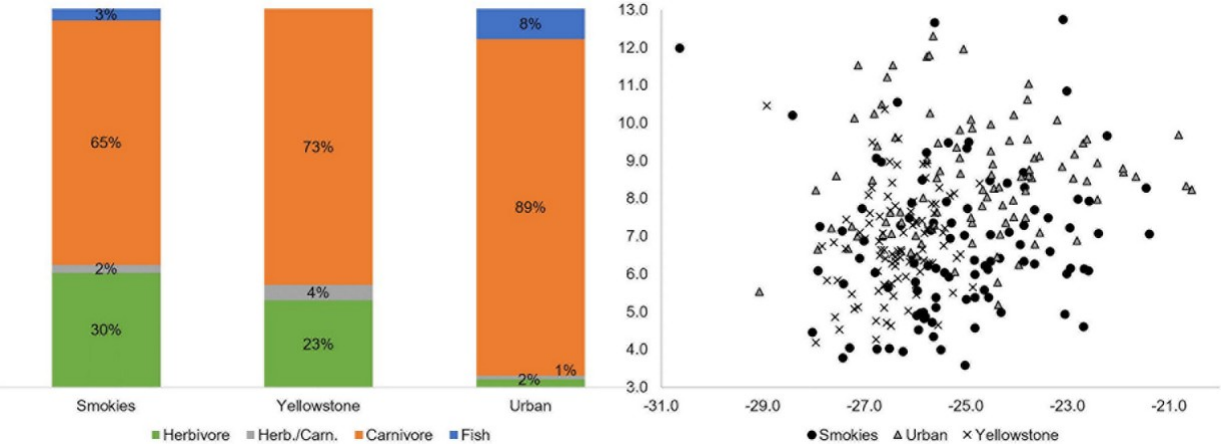
Putative carcass trophic values transformed from wild fly data by mean experimental fractionation factors for females. Panel A: Proportion (out of 100%) of wild flies from urban, Smokies, and Yellowstone regions with isotopic signatures indicative of four carcass trophic positions: herbivore (green), herbivore/carnivore (grey), carnivore (orange), fish (blue). Panel B: Summary of results from wild flies are illustrated via scatterplot with δ^15^N given on the y-axis and δ^13^C values on the x-axis. Black circles = flies from the Great Smoky Mountains National Park; grey triangles = flies from the urban environment (Indianapolis, IN, USA); black X’s = flies from Yellowstone National Park.

Overall, carcass trophic structure was significantly different between the urban environment and both national parks (*P* < 0.001; Fig 4). The national parks had greater similarity in trophic structure, with approximately three quarters of flies exhibiting carnivore signatures, approximately one quarter exhibiting herbivore signatures, and few exhibiting signatures indicative of fish. Conversely, 90% of flies from the urban environment had detectable carnivore carcass signatures, with the remainder of these flies mostly exhibiting signatures of fish.

## Discussion

This study has demonstrated that blow flies can be used as an informative surveillance tool for monitoring terrestrial vertebrate communities. Using our experimentally derived isotopic fractionation data, we were able to infer the broad vertebrate trophic level structure present in three different environments of varying degrees of human influence: Yellowstone National Park, a pristine environment; the Great Smoky Mountains National Park, a protected area that is also the most visited national park in the United States; and the greater Indianapolis area, which is an urban environment boasting a population of almost 2 million people. Specifically, we were able to determine what types of carcasses our adult flies used for their prior larval development, which cannot be done with DNA analysis of the adults as the carcass DNA is broken down during metamorphosis. By comparing data from the current SIA study to our previous molecular DNA analysis of the same flies, we have demonstrated that blow flies utilize primarily carnivore carcasses for larval development and primarily herbivore resources (either carcasses or feces) for adult consumption in the three environments sampled. We provide evidence that SIA is a powerful method for gleaning information about what these insects were doing before their metamorphosis into adults. With careful experimentation, we were able to demonstrate a predictable δ^15^N fractionation in adult flies compared to their larval diet that does not appear to vary significantly by adult diet or by the aging process of the adult fly.

The overall isotopic fractionation of female blow flies from our laboratory experiments aligns with similar values compiled from the extensive body of animal isotopic literature [27], lending more confidence to deriving carcass trophic diversity metrics from these flies. The trophic positions of carcasses given here align with much of the molecular and chemical data from the same flies previously reported [12]. Vertebrate DNA data from flies in the urban environment showed a greater proportion of medium sized carnivores (e.g., dogs and raccoons) visited by adult flies compared to flies collected in the national parks, and herbivores (e.g., white-tailed deer, groundhogs, eastern cottontails) made up a much smaller proportion of animals detected from molecular analysis of urban flies. The molecular data is supported by the isotopically-derived data presented here with overall higher δ^15^N values observed in flies from the urban environment compared to the other regions. This may be attributed to a higher number of carnivore carcasses available due to road mortality events caused by humans. Indianapolis is the 13^th^ largest city in the United States [28] with 20,000 km of roadways [29]. Subsampling of roadkill deaths from 9 locales in Indiana from 2005 to 2006 showed 457 mammal mortality events, with 39% and 32% of those deaths made up of Virginia opossums and raccoons, respectively [30]. This aligns with both molecular and isotopic data from the current study, as both opossums and raccoons made up a large portion of vertebrate DNA derived from flies in the urban environment, and they may have potentially served as a portion of the ~90% of carnivores utilized by flies for larval development [12]. Conversely, the DNA-derived vertebrate composition of the national parks contrasted with isotopic data, suggesting that carnivore carcasses are utilized more often for larval development and herbivore resources (e.g., feces) are used more often for adult survival [14, 31]. Given the sheer abundance of herbivore populations in the national parks, we initially expected a proportional number of flies collected in these environments to exhibit herbivore carcass signatures. However, we did not account for the presence of numerous vertebrate predators and scavengers in the national parks: coyotes, black bears, eagles, and vultures in the Smokies; black bears, grizzly bears, wolves, ravens, and eagles in Yellowstone. The presence of these scavengers likely introduces strong intra-guild competition with blow flies vying for opportunities to exploit carcasses. In Yellowstone specifically, wolves provision other vertebrate scavengers with carcasses from their hunts, most of which are herbivores (e.g., elk) [32]. Some vertebrate scavengers, like ravens and coyotes, track wolf packs in order to exploit wolf-kills [33, 34]. In order for blow flies to “win” this inter-class competition, they must first locate the carcass and successfully colonize it before other scavengers arrive, with their ensuing eggs, larvae, and pupae constantly facing the threat of predation and death by larger animals. Compared to highly sought-after herbivores, the relatively low abundance of predators and scavengers in these environments may give flies an advantage for exploiting carnivore carcasses simply due to decreased competition. In fact, some studies suggest that vertebrate scavengers actively avoid consuming carnivore carcasses, or at least conspecifics, as scavenging these resources may be associated with a greater likelihood of exposure to infectious diseases [35]. This avoidance strategy by vertebrate scavengers has also been shown to facilitate greater opportunities for invertebrate scavengers to exploit carnivore carcasses [36], which likely explains the carnivore bias in our blow flies collected in the national parks.

Higher δ^13^C values were observed more in flies from the urban and Smokies environments compared to flies from Yellowstone. This could likely be an influence of corn-based foods (generated by humans) being more available in this environment. Domestic or agricultural animals being fed or supplemented with corn-based diets make up a portion of the animals detected by DNA in these areas (e.g., cows, pigs, chickens), therefore their muscle tissues may be more enriched in ^13^C assimilated from their C_4_ (e.g., corn-based) diet. Additionally, opportunistic vertebrate scavengers (e.g., raccoons, opossums, black bears) may also have access to human junk food, fast food (often deep-fried in corn oil, [37]), and corn-based food waste that could have the same isotopic effect on their tissues. These animals are the likely vectors for introducing corn-based diets into the fly tissues. If flies develop on the tissues of any of these animals, their δ^13^C isotopic signatures would more likely resemble those of corn-fed animals. Conversely, flies collected in Yellowstone had lower δ^13^C values compared to the other regions, which indicates that the herbivores they fed upon were potentially consuming a mixture of C_3_ and C_4_ plants.

Unlike nitrogen, there was a consistent sex-effect on carbon fractionation with female flies almost always exhibiting lower δ^13^C values compared to males. This could be explained by quicker turnover rates that might be occurring in female flies throughout their lifetimes as compared to males. Isotopic turnover can vary among different tissues within an individual, e.g., lipids that tend to have low δ^13^C (1–2 ‰) relative to body tissue [38]. This would make sense given that female blow flies have a higher fat content on average compared to males [39]. Larvae of other fly species, like *Chironomus acerbiphilus* (Diptera: Chironomidae), can exhibit increased δ^13^C during periods of starvation due to the preferential breakdown of body tissue (e.g., muscle) and respiration of ^12^CO_2_ when dietary carbon is unavailable [40]. Thus, sex differences in δ^13^C seen in this study could be due to the breakdown of previously assimilated ^12^C from the larval diet in the females, perhaps due in part to reproductive needs of the maturing female flies. Conversely, males may assimilate ^13^C quicker than females, leading to the differences observed (males with higher δ^13^C). Additionally, males and females may metabolize components that have different δ^13^C values altogether. Regardless of the exact mechanism, it will be important for future studies to take sex into consideration when refining interpretations from carbon isotopic data derived from flies.

We also provide evidence that, for the most part, the adult diet does not alter the larval diet isotopic signatures in adult flies. This means that the carbon and nitrogen isotopic signatures obtained from flies can be reliably attributed to the fly’s larval resource and not environmental or physiological conditions experienced by the adult fly itself. We also showed that fly age does not affect the δ^15^N signatures in blow fly tissues for up to 15 days (an average lifespan of a wild fly is three to four weeks, [41]); however, δ^13^C increases as the fly gets older, and this is exacerbated if the fly has taken a protein meal during adult life. Other female holometabolous insects have shown similar decreases in δ^13^C throughout their adult lifetimes caused by rapid isotopic turnover in reproductive organs and fatty tissue [42]. The δ^13^C of invertebrate tissues can evolve with time as a result of metabolic activity combined with changes in dietary sources that have distinct isotope compositions [37]. Therefore, the significant enrichment in our chicken-fed flies over time may be due to the switch in diet from chicken to sugar and water for the remainder of the fly’s lifetime, as well as isotopic turnover in the tissues. Though this does represent an effect of adult diet on the isotopic signature of the fly, it is likely not seen in nature where flies have access to multiple protein sources (including carrion, refuse, and feces) throughout their lifetime.

Though the experiments performed in this study specifically targeted the effects of feeding and age on isotopic signatures, other intrinsic variables, such as fly size, were not explicitly investigated but could prove especially informative for this method. The size of the adult fly is influenced by the amount of food consumed as a larva, i.e., larger larvae = larger adults. Therefore, the density of larvae on a resource can significantly affect their size, and crowded resources (e.g., carcasses) tend to produce smaller adults due to larval competition [43]. Additionally, it is common that female blow flies are larger on average than males [44]. Therefore, there may be a sex, larval size, and carcass size effect on δ^15^N signatures in adult flies that should be accounted for if using flies to ascertain carcass trophic positions of a terrestrial environment. For example, the average carbon isotope fractionation for the diet experiments exhibited changes in magnitude and direction that ranged from -2.9‰ to 2.5‰. For each experiment, food sources were well-controlled and isotopically characterized, although the amount of sugar uptake by individual flies is unknown. The size of the rearing containers limited the number of larvae to ~150–200 and therefore mitigated resource limitations or density effects. Although it is unclear whether the variance observed in carbon isotope fractionation is environmental or intrinsic to the fly metabolism, the range of ~ ± 3‰ is small relative to the distinct and non-overlapping δ^13^C values of C_3_ or C_4_ plant-based carbon sources [18, 22] that would be available to temperate-zone biomes.

When used in conjunction with other analytical methods, SIA can add another rich dimension to community assessment survey methods using carrion insects. Today’s changing climate requires special tools to assess community diversity that can be quickly implemented and be as minimally invasive as possible to maintain the integrity of fragile ecosystems. The use of SIA of necrophagous insects, like blow flies, allows researchers to gain a snapshot of the overall vertebrate trophic level structure of an ecosystem with little disturbance. Simultaneous use of isotopic, molecular, and chemical data derived from flies in these areas can give a quick, comprehensive survey of vertebrate communities that rivals any traditional survey method currently being used. Additionally, SIA of blow flies allows researchers to assess the dead vertebrate community in a way that is difficult to accomplish using other techniques, including molecular methods. As most blow fly larvae are limited to developing on dead animals, isotopes reveal carcasses that are available in an environment, with no additional resources (e.g., feces) confounding interpretations.

## Materials and methods

### Study organism

Most blow flies are carrion-breeding insects that require a dead animal for development [45]. The life cycle of the blow fly begins when a gravid adult female locates a carcass and lays her eggs on it. After the eggs hatch, the larvae proceed through successive development stages as they continue to feed and grow larger in size. Once a critical weight threshold is surpassed [46], larvae wander away from the carcass in order to pupate in the soil. After the puparium is formed, the insect metamorphoses into an adult fly. After one to two weeks, the adult fly emerges from the puparium and begins the “free-roaming” portion of its life cycle. As an adult, the female blow fly will utilize carrion and vertebrate feces for protein [31, 47, 48], as well as nectar or other sugary resources for carbohydrates [49, 50]. For this study, the black blow fly *Phormia regina* was chosen as the study organism due to its prevalence in the environment as well as the ease at which it can be maintained in a laboratory colony.

### General procedure for feeding experiments

Laboratory colonies (<G_5_) generated from wild-caught *P*. *regina* were used for all experiments. When not being utilized for experimental purposes, flies were maintained at ambient conditions (~22°C, 50%RH) in the “fly room” at IUPUI, Indianapolis, Indiana, USA and given water and table sugar *ad libitum*. Adult flies were aged 3 to 4 d post-eclosion before being used in any feeding experiment (i.e., they were not given any protein resources during this time). To ensure that each fly had an equal opportunity to land on and taste the resource, flies were always individually placed in sterile, clear medicine cups with 1g of the respective resource and a Kimwipe soaked in distilled water (negative control flies were only exposed to a water-soaked Kimwipe). Cups were then randomized and placed inside of a Percival I-36VL incubator (Percival Scientific Inc., Perry, IA, USA) at 28°C and 65% RH. Exposure time lasted 4 h for all adult feeding experiments to ensure ample opportunities for the fly to taste the resource. Flies were then released from the feeding cups into a sterile container (either a cage or a sterile mosquito breeder; one for each treatment group) with water available until flies were killed.

### Experiments

#### Effects of preservation

This experiment was designed to investigate if preservation method of the flies alters the primary isotopic composition of fly tissue. This is relevant as flies that are collected in the field are usually killed in ethanol, while flies used in lab experiments are often frozen. A single colony of *P*. *regina*, reared on turkey breast to adulthood, was used to investigate this question. Cohorts of unfed adult males (N = 10) and females (N = 10) were killed by freezing at -20°C (i.e., “frozen flies”) or by submerging in 95% ethanol (i.e., “ethanol flies”), for a total of N = 20 flies per treatment.

#### Persistence of larval diet isotopic signature

The second experiment was designed to determine if larval resources could be differentiated based on the isotopic signature of the adult flies. Three *P*. *regina* colonies were reared on either beef liver, chicken liver, or salmon filets. Colonies were allowed to complete one generation on the respective resource prior to the current experiment. Flies (N = 5 males, N = 5 females per colony) were not given a protein resource as adults, but were only exposed to water and table sugar, *ad libitum*, and then killed 4 d post-eclosion.

#### Effect of single adult feeding event

The third experiment was designed to determine if a larval isotopic signature persists following adult feeding. Colonies of *P*. *regina* were reared on beef liver and then subjected to a feeding experiment in which adult cohorts (N = 9 male, N = 9 female per treatment) were individually exposed to 1g of either beef liver, salmon filet, or carnivore feces (lion, *Panthero leo* Linnaeus) to test whether adult feeding affects the detection of larval isotopic signatures. A cohort of unfed flies were also tested as negative controls. Flies were allowed to feed for 4h then killed using the freezing method.

#### Time series

The fourth experiment focused on determining if the larval resource isotopic signature is maintained throughout the duration of the adult life. One *P*. *regina* colony was reared on salmon filet for a single generation. Upon eclosion, flies were released into a clean cage and given water and sugar *ad libitum*. When the flies were ~3d old, they were subjected to a feeding experiment in which half were selected as a negative control cohort (“unfed”) and the remaining half were exposed individually to 1g chicken liver (using methods outlined above for feeding experiments; these flies are referred to as “fed”). For each treatment, a sample size of N = 10 (5 males, 5 females) was used per sampling event. Flies were killed 4h, 3d, 9d, and 15d after the conclusion of the exposure period.

### National park and urban samples

In order to determine the utility of using isotopic analysis of flies to characterize the carcass trophic level structure in the wild, we sampled wild blow flies in three regions: 1) Indianapolis, Indiana (“urban”), 2) the Great Smoky Mountains National Park (“Smokies”), and 3) Yellowstone National Park (“Yellowstone”), as previously reported [12]. The permits to sample in the national parks were obtained via application through the National Park Service Research Permit and Reporting System: Great Smoky Mountains: Permit # GRSM-2018-SCI-2036, study # GRSM-02039; Yellowstone National Park: Permit #YELL-2018-SCI-8046, study # YELL-08046). All locations in the parks were authorized and included areas close to roads/parking areas and away from developed areas. Sampling occurred at N = 4 separate timepoints from June to August at each of N = 4 different geographic sites per region (Table 4). Sampling in the urban environment occurred in 2016 while sampling in the national parks occurred in 2018. Flies were morphologically identified and sorted so that only females of *P*. *regina* were used. We used a random number generator to select a maximum of N = 10 random females from each individual spatiotemporal sample. Selected flies that underwent DNA [12] and fecal [14] analysis were also subjected to SIA (urban, N = 100; Smokies, N = 96; Yellowstone, N = 98).

**Table 4:** Summary of blow fly sampling sites per region.

Region	Site	City, State	Coordinates	Flies Analyzed (proportion)
Urban	Military Park	Indianapolis, IN	39.771, -86.169	20 (30.3%)
	Northwest Park	Greenwood, IN	39.629, -86.144	30 (30.9%)
	Skiles Test Park	Indianapolis, IN	39.868, -86.049	20 (12.3%)
	University Park	Greenwood, IN	39.611, -86.051	30 (28.3%)
Smokies	Site 1	near Gatlinburg, TN	35.735, -83.413	20 (13.6%)
	Site 2	near Gatlinburg, TN	35.704, -83.365	20 (13.0%)
	Site 3	near Gatlinburg, TN	35.663, -83.526	26 (17.1%)
	Site 4	near Gatlinburg, TN	35.671, -83.680	30 (5.2%)
Yellowstone	Site 1	near Gardiner, MT	44.614, -110.414	26 (39.4%)
	Site 2	near Gardiner, MT	44.958, -110.542	30 (22.6%)
	Site 3	near Gardiner, MT	44.958, -110.312	22 (32.4%)
	Site 4	near Gardiner, MT	44.886, -110.144	20 (37.7%)

Urban = Indianapolis, IN, USA, Smokies = Great Smoky Mountains National Park, Yellowstone = Yellowstone National Park. Only female P. regina were selected for analyses. The last column refers to the number of flies analyzed per site, as well as the proportion of flies analyzed out of the entire sample (in parentheses).

### Isotope ratio mass spectrometry

To prepare for isotopic analysis, flies from all experiments and field work were decapitated and heads were desiccated in a drying oven set at 40°C for 24 h. We analyzed fly heads for the simplicity of sample preparation and because the relatively slow isotopic turnover rates observed in cuticle relative to other tissue suggests these body parts retain a longer-term isotopic record of diet [30]. Comparison between the δ^15^N of the head and abdomen of a male and female fly were within 0.3‰. Each fly head was then placed inside of a 9mm x 5mm tin capsule and weighed on a microbalance. All samples were combusted in an EAIsoLink elemental analyzer coupled under continuous helium flow to a Thermo DeltaV Plus IRMS. Sample values were corrected to the international reference materials, whose isotopic values bracket the range of the samples. Sample δ^15^N values were calibrated relative to Air by linear regression to USGS-40 L-glutamic acid (U.S. Geological Survey, δ^15^N = -4.52‰), USGS-41 L-glutamic acid (U.S. Geological Survey, δ^15^N = 47.51‰), and Bovine Liver RM 1577c (National Institute of Standards and Technology, δ^15^N = 8.14‰). Sample δ^13^C values were reported relative to Vienna Pee Dee Belemnite by linear regression of USGS-40, δ^13^C = -26.39‰; USGS 41, δ^13^C = -37.63‰; Bovine Liver RM 1577c, δ^13^C = -17.78‰. The following equation was used:

δ^X^E = [(R_Sample_/R_Standard_)– 1], where ^x^E = ^15^N or ^13^C and R = ^15^N/^14^N or ^13^C/^12^C. Analytical precision for δ^15^N and δ^13^C was ±0.2‰, based on replicate standard analyses.

### Larval resource (carcass) trophic assignment

In order to deconvolute the isotopic signatures observed in wild flies, it was necessary to gather pertinent wild vertebrate data that would correspond to the isotopic composition of the flies. Unfortunately, no isotopic repository exists for such an endeavor, so an analysis of isotopic data available from pertinent literature reviews was performed to obtain δ^15^N values from animals within broad trophic categories: carnivore [15, 51–55], herbivores [15, 56, 57], and fish [15, 56]. Only δ^15^N values were used as this is the primary isotope used for trophic level assignment. Criteria for this analysis included data obtained only from animal subjects exhibiting a Nearctic distribution (e.g., African wildlife were excluded, which represents a large portion of available isotopic data from wild vertebrates), and the isotopic values must have originated from either blood or muscle tissue as this is the primary food source for blow fly larvae.

### Statistical analyses

The statistical program R was used for all statistical analyses [58]. A multivariate analysis of variance (MANOVA) was used to analyze data from the experiments. Additionally, a univariate ANOVA was used as a post-hoc test when significant effects were observed in the MANOVA. For the preservation experiment, carbon and nitrogen isotopic values were used as the dependent variables, whereas fractionation was used as dependent variables in all other experiments. Treatment and fly sex were used as independent variables in each experiment. Though a MANOVA was implemented with the Time Series experiment data as an exploratory analysis step, a Kruskal-Wallis test with post-hoc Dunn’s test [59] were also performed since some of the data from this experiment were not normally distributed (Shapiro-Wilk, *P* < 0.05). Additionally, “unfed” and “fed” data from this experiment were analyzed together first, then separately so as to determine the specific changes in isotopic fractionation over time depending on whether or not flies had taken a meal. Finally, a Kruskal-Wallis test with post-hoc Dunn’s test were also used to analyze the wild fly data in order to statistically differentiate between locations.

Isotopic values of N = 10 different carnivores, N = 15 different herbivores, and N = 13 different fish from the literature were analyzed to determine if they could be statistically differentiated for use as the trophic categories in our wild fly analysis. An ANOVA with post-hoc Tukey’s honestly significant difference (HSD) was performed on nitrogen isotopic values to determine which trophic categories could be statistically differentiated. Once the trophic categories were chosen based on their statistical differentiation, likelihood ratios were generated in Excel to assign a probability of trophic category to each nitrogen value. Methods for probability distribution generations followed those outlined in Picard and Wells (2012) [60]. Thus, specific trophic categories and ranges for δ^15^N were established that were then used to determine carcass trophic position from wild fly isotopic signatures. Experimental enrichment and depletion data averaged from all experiments for female flies specifically were used to transform raw wild fly data in order to approximate the carcass trophic position from each wild fly. Finally, with the trophic categories derived from the literature, wild flies were assigned into putative carcass trophic positions.
